# Plasma proteome responses in zebrafish following λ-carrageenan-Induced inflammation are mediated by PMN leukocytes and correlate highly with their human counterparts

**DOI:** 10.3389/fimmu.2022.1019201

**Published:** 2022-09-29

**Authors:** Ives Charlie-Silva, Natália M. Feitosa, Leticia G. Pontes, Bianca H. Fernandes, Rafael H. Nóbrega, Juliana M. M. Gomes, Mariana N. L. Prata, Fausto K. Ferraris, Daniela C. Melo, Gabriel Conde, Letícia F. Rodrigues, Mayumi F. Aracati, José D. Corrêa-Junior, Wilson G. Manrique, Joshua Superio, Aguinaldo S. Garcez, Katia Conceição, Tania M. Yoshimura, Silvia C. Núñez, Silas F. Eto, Dayanne C. Fernandes, Anderson Z. Freitas, Martha S. Ribeiro, Artem Nedoluzhko, Mônica Lopes-Ferreira, Ricardo C. Borra, Leonardo J. G. Barcellos, Andrea C. Perez, Guilheme Malafaia, Thiago M. Cunha, Marco A. A. Belo, Jorge Galindo-Villegas

**Affiliations:** ^1^ Department of Pharmacology, University of São Paulo, São Paulo, Brazil; ^2^ Integrated Laboratory of Translational Bioscience, Institute of Biodiversity and Sustainability, Federal University of Rio de Janeiro, Macaé, Brazil; ^3^ Department Immunology, University of São Paulo, São Paulo, Brazil; ^4^ Laboratório de Controle Genético e Sanitário, Faculdade de Medicina Universidade de São Paulo, São Paulo, Brazil; ^5^ Reproductive and Molecular Biology Group, Department of Morphology, Institute of Biosciences, São Paulo State University, São Paulo, Brazil; ^6^ Transplantation Immunobiology Lab, Department of Immunology, Institute of Biomedical Sciences, Universidade de São Paulo, São Paulo, Brazil; ^7^ Department of Pharmacology, Instituto de CiênciasBiomédicas-Universidade Federal de Minas Gerais (ICB-UFMG), Belo Horizonte, Brazil; ^8^ Department of Pharmacology and Toxicology, Oswaldo Cruz Foundation (FIOCRUZ), Rio de Janeiro, Brazil; ^9^ Laboratory of Zebrafish from Federal de Minas Gerais (UFMG), Belo Horizonte, Brazil; ^10^ Department of Preventive Veterinary Medicine, São Paulo State University, São Paulo, Brazil; ^11^ Department of Morphology, Instituto de CiênciasBiomédicas-Universidade Federal de Minas Gerais (ICB-UFMG), Belo Horizonte, Brazil; ^12^ Veterinary College, Federal University of Rondonia, Rolim de Moura, Brazil; ^13^ Department of Aquaculture, Faculty of Biosciences and Aquaculture, Nord University, Bodø, Norway; ^14^ Department of Lasers in Dentistry, São Leopoldo Mandic, Campinas, Brazil; ^15^ Peptide Biochemistry Laboratory, Universidade Federal de São Paulo (UNIFESP), Sao Jose Dos Campos, Brazil; ^16^ Center for Lasers and Applications, Instituto de PesquisasEnergéticas e Nucleares (IPEN-CNEN), Sao Paulo, Brazil; ^17^ University Brazil, São Paulo, Brazil; ^18^ University Brazil, Descalvado, Brazil; ^19^ Development and Innovation Laboratory, Center of Innovation and Development, Butantan Institute, São Paulo, Brazil; ^20^ Paleogenomics Laboratory, European University at Saint Petersburg, Saint Petersburg, Russia; ^21^ Immunoregulation Unit, Butantan Institute, São Paulo, Brazil; ^22^ Department of Genetics and Evolution, Federal University of São Carlos, São Paulo, Brazil; ^23^ Postgraduate Program in Pharmacology, Federal University of Santa Maria, Rio Grande do Sul, Brazil; ^24^ Postgraduate Program in Bioexperimentation. University of Passo Fundo, Rio Grande do Sul, Brazil; ^25^ Biological Research Laboratory, Goiano Federal Institute, Urutaí, Brazil; ^26^ Center of Research in Inflammatory Diseases, Ribeirão Preto Medical School, University of São Paulo, São Paulo, Brazil; ^27^ Department of Pharmacology, Ribeirão Preto Medical School, University of São Paulo, São Paulo, Brazil; ^28^ Department of Genomics, Faculty of Biosciences and Aquaculture, Nord University, Bodø, Norway

**Keywords:** acute-phase proteins, *Danio rerio* (zebrafish), glycoproteins, immunity, model organism, optical coherence tomography (OCT), proteomics, shotgun LC-MS/MS

## Abstract

Regulation of inflammation is a critical process for maintaining physiological homeostasis. The λ-carrageenan (λ-CGN) is a mucopolysaccharide extracted from the cell wall of red algae (*Chondrus crispus*) capable of inducing acute intestinal inflammation, which is translated into the production of acute phase reactants secreted into the blood circulation. However, the associated mechanisms in vertebrates are not well understood. Here, we investigated the crucial factors behind the inflammatory milieu of λ-CGN-mediated inflammation administered at 0, 1.75, and 3.5% (v/w) by i.p. injection into the peritoneal cavity of adult zebrafish (ZF) (*Danio rerio*). We found that polymorphonuclear leukocytes (neutrophils) and lymphocytes infiltrating the ZF peritoneal cavity had short-term persistence. Nevertheless, they generate a strong pattern of inflammation that affects systemically and is enough to produce edema in the cavity. Consistent with these findings, cell infiltration, which causes notable tissue changes, resulted in the overexpression of several acute inflammatory markers at the protein level. Using reversed-phase high-performance liquid chromatography followed by a hybrid linear ion-trap mass spectrometry shotgun proteomic approach, we identified 2938 plasma proteins among the animals injected with PBS and 3.5% λ-CGN. First, the bioinformatic analysis revealed the composition of the plasma proteome. Interestingly, 72 commonly expressed proteins were recorded among the treated and control groups, but, surprisingly, 2830 novel proteins were differentially expressed exclusively in the λ-CGN-induced group. Furthermore, from the commonly expressed proteins, compared to the control group 62 proteins got a significant (*p* < 0.05) upregulation in the λ-CGN-treated group, while the remaining ten proteins were downregulated. Next, we obtained the major protein-protein interaction networks between hub protein clusters in the blood plasma of the λ-CGN induced group. Moreover, to understand the molecular underpinnings of these effects based on the unveiled protein sets, we performed a bioinformatic structural similarity analysis and generated overlapping 3D reconstructions between ZF and humans during acute inflammation. Biological pathway analysis pointed to the activation and abundance of diverse classical immune and acute phase reactants, several catalytic enzymes, and varied proteins supporting the immune response. Together, this information can be used for testing and finding novel pharmacological targets to treat human intestinal inflammatory diseases.

## Introduction

Despite enormous advances in the understanding of tissue organization, homeostasis, and inflammation in vertebrates, their study is of growing interest among immunologists. Mainly, inflammation is a complex biological response of body tissues to potentially harmful stimuli, including pathogens and physiological tissue damage (1). However, throughout the inflammatory process, the principles of organization between the physiological and altered state of immune cells and the resulting molecular mediators at gene and protein level remain poorly understood. Moreover, understanding the regulatory mechanisms from acute to chronic inflammation is critical but so far only few studies have been performed on higher vertebrates (2).

Carrageenan (CGN) is a polysaccharide extracted from red algae (*Chondrus crispus*) (3). It is a high molecular weight sulfated linear galactan whose basic structure results from the 3,6-anhydrogalactose content, location, and number of sulfates per group (4). The mucopolysaccharides of CGN are structured as anionic linear polymers composed of 1,3α-1,4β-galactans with one kappa (κ-), two iotas (ι-), or three lambda (λ-) sulfates per disaccharide unit. The hybrid nature resulting from the action of CGN at the molecular level is responsible for the changes in the physical, chemical, and biological properties of each preparation (5). The ι- and λ-CGN have a higher inflammatory potential than the κ- fraction. And, the higher number of sulfated sugars present in λ-CGN are responsible for the activation of inflammatory mediators and the production of vascular and cellular events of inflammation (6).

Many *in vitro* studies using human intestinal epithelial primary cells or colorectal and ileal adenocarcinoma cell lines have reported activation of Wnt/ß-catenin signaling or NF-κB inflammatory pathway and inhibition of apoptosis following induction by λ-CGN as the inflammatory agent (7, 8). Moreover, studies in animal models consistently report that λ-CGN induces histopathological features resembling those of human inflammatory bowel disease (IBD), disrupts the intestinal epithelial barrier, inhibits immune pathways that protect against noxious antigens, and stimulates the release of several proinflammatory cytokines (9–12). Moreover, studies using serum of λ-CGN treated animals observed highly reproducible cellular influx and inflammatory exudate and converged on a limited set of proteins that are repeatedly identified but represent only a small fraction of the entire blood proteome (13, 14). However, a comprehensive overview of how the immunomodulatory activity of λ-CGN affects the plasmatic protein landscape in vertebrates has yet to be fully elucidated.

The development and advancement of “omics” technologies have revolutionized the field of immunology and inflammation. Among these, transcriptomic studies the transcriptome, which provides an overview of RNA molecules required to interpret the functional elements of the genome and understand patterns of development and disease (15). Although transcriptomics is considered a robust tool, proteomic studies provide more valuable information, as changes in various biological pathways, including molecular function and cellular assembly, are supported by a large-scale study of proteomes (16). The proteome is the complete set of proteins expressed by an organism or specific tissue. Proteins are a vital part of living organisms, as they are the main component of the physiological and metabolic pathways. Therefore, protein-protein interaction networks and proteome profiling through different methods allow the identification of new biomarkers, disease related pathways and reveal essential proteins involved in adaptive and pathological processes more accurately than transcriptomics, as the proteome provides information beyond the messenger RNA expression profile of a particular genome (17). For example, in humans, active intestine inflammation is associated with an acute phase reaction and migration of leukocytes to the intestine, which is translated into the production of several acute phase proteins (APPs), which may be easily and reliably detected in serum or plasma (18, 19). Analytical choices range from classical two-dimensional gel electrophoresis and mass spectrometry to sophisticated nanofluidic chromatography and computational inferences (20, 21). However, regardless of the method selected, reliable exploration of physiological functions of proteins and their systemic interactions *via* the blood as a transporter between organs requires the context of a whole organism. Therefore, animal models have become very popular for obtaining experimental samples that mimic the desired condition or disease under research (22).

The first challenge to overcome is the selection of appropriate animal models to achieve the desired goal. Vertebrate animal models have proven their efficacy in screening inflammatory responses and determining specific molecular therapeutic targets in human medicine (23–25). In this context, zebrafish (ZF) emerged as an outstanding genetically tractable and practical model for screening the biological process of inflammation *in vivo (* 26, 27). Furthermore, this fish is a very versatile model organism. Following proper automated settings, ZF of various genetic backgrounds can be visually inspected from specific single-cell elements using transmitted light or fluorescent imaging (28). Simultaneously, the signaling pathways and chemical interactions that regulate the development and progress of inflammation in any tissue can be analyzed in exquisite detail (29–31). Furthermore, whole genome sequencing and emerging data have shown that the physiology and immunity of ZF present specializations parallel to mammals, making this small fish a widely used model for the study of human diseases (32). Consequently, many ZF transgenic lines have been developed that recapitulate multiple models of human inflammatory diseases (33–36).

In particular, in recent years, several ZF models recapitulating intestinal inflammatory processes expressed or observed in various vertebrate organisms, from fish to humans have received much attention (37, 38). The coelomic cavity of zebrafish (according to a recent study (39), henceforth the peritoneal cavity) shares many similarities with the human gastrointestinal system. It has comparable genetic networks, similar anatomy, physiology, as well as absorptive and secretory functions (40). Moreover, the collection of characteristics and similarities are inducible and prone to manipulation following classical and state-of-the-art settings. Thus, building on these previous studies, we set out to validate a model of λ-CGN intraperitoneal inflammation in ZF while identifying whether a resulting activation of immunocytes leads to the generation of inflammatory and immune pathways that, in turn, may influence the role of plasma protein levels in treated animals. From the results obtained, bioinformatic analyses were performed to reveal the plasma proteome and undertake 3D model reconstructions to demonstrate that ZF is a convincingly overlapping model of the human-associated inflammatory proteins that provides a valuable preclinical model for the development of future therapies.

## Material and methods

### Animal care and maintenance

All animal experimentation protocols were performed following the Brazilian animal welfare legislation (CONCEA N° 34, 27/07/2017 - MCTI) and approved by the Bioethical Committee of the University UFMG (approval CEUA-UFMG 336/2017). In particular, the number of animals used in the trial was determined following a highly restricted *f* size *a priori* effect established at the 0.05 α-error probability on the Power analysis accomplished with the GPower software (41). Adult (6 months-old) male zebrafish (*Danio rerio*) of the AB wild-type strain (1.0 ± 0.07 g) were used for the experiment. The fish were obtained locally from the Aquaculture Laboratory, Veterinary School of the Federal University of Minas Gerais, Brazil. Importantly, only male fish were used to avoid intrinsic bias resulting from disparities in ventral size resulting from mixed sex groups. The fish were maintained in a filtered recirculating water aquarium system at 28.5°C with a 14h light and 10h dark photoperiod and standard husbandry conditions (42). The animals were fed commercial food pellets (Gemma micro, Skretting, USA) twice daily, *ad libitum*.

### Carrageenan induction assays

The assessed treatments consisted of two carrageenan prepared injectable solutions and PBS as sham control. Adult male zebrafish individuals were fed, weighed, and injected with a λ-CGN, type IV (Cat. #22049; Sigma, USA) containing solution at 1.75 and 3.5% dissolved in PBS according to weight of each individual fish (27). Briefly, adult zebrafish contained in each test group were fed, sedated, and intraperitoneally (i.p.) injected with 30 μl of the corresponding treatment solutions using an Ultra-Fine™ BD syringes (Beckton & Dickinson, Brazil), with a 6 mm (5/16”) needle and 0.25 mm (31 G) caliber. Meanwhile, some fish were injected with phosphate-buffered saline (PBS) with pH 7.28 at 4°C to serve as the control (sham-exposed) group. After induction, the fish in each group were monitored and sampled (n=6; 3 fish per group in duplication) every hour for a 7 h period as previously described (43).

### Optical coherence tomography, procedures and assessment

Four hours post-injection with 3.5% λ-CGN, the treated and control zebrafish were immobilized and mounted in a Petri dish in horizontal and ventral positions. The OCT scanning was performed with the OCP930SR system (Thorlabs Inc., USA). Infrared reflection images (λ = 930 nm) were recorded with 100 nm spectral bandwidth, providing a resolution of 6 mm in air. The images were acquired with 512 vs. 2000 pixels (depth vs. lateral). The distance between the sample and the OCT probe was kept constant (~25 mm) during all trials. The OCT probe was positioned to visualize the maximum aperture of the skin. This procedure was adopted to allow the assessment of the same sites. For three-dimensional image reconstruction (3D), a precise displacement of the sample in the y-axis (orthogonal to the image acquired by the OCT system) during the image acquisition was done by a computer-controlled linear translation stage (T25-XYZ, Thorlabs Inc., USA) with a minimum step of 0.05 μm. The 3D images were reconstructed with VGStudioMax (version 1.2; Volume Graphics, Heidelberg, Germany) and analyzed with Image J (National Institutes of Health, Bethesda, Maryland., USA).

### Peritoneal exudate collection and leukocyte analysis

Peritoneal leukocyte exudates were collected from sedated zebrafish injected i.p. with 50 µL PBS+ 3% Albumin + 3% Heparin solution at each selected sampling point. Briefly, the fish abdomen was massaged for 2 min to dislodge tissue-attached cells into the PBS solution. A micro-incision was then made below the lateral line to access the peritoneum, and peritoneal exudates were aspirated and collected in a 1.5 ml microcentrifuge tube (Eppendorf, Hamburg, Germany). Total cell count were performed using a flow cytometer at a rate of 10,000 events/s (BD FACSCalibur™, Becton Dickinson, Brazil). Results are presented as the number of cells per cavity. All FACS parameters (FSC and SSC) and region settings were gated following the same settings throughout the different experiments. Analyses were performed with FlowJo software (TreeStar, USA). Besides, aliquots of cell suspensions were loaded into Cytospin (Thermo Fisher Scientifics, USA) chambers, centrifuged at 200*g* for 2 minutes, fixed, and stained with May Grunwald following standard protocols. After staining, the cell types were identified using light microscopy and standard morphological criteria.

### Whole mount histopathological assessment

Necropsy was performed to evaluate histological changes produced by λ-CGN induced inflammation. Briefly, fish (n=30) per treatment were collected and fixed in 10% neutral buffered formalin for 72h at room temperature. All specimens were then decalcified in 0.5 M Ethylenedinitrilo-tetraacetic acid for 48h (44). The decalcified fish were then processed for routine paraffin axial embedding, sagittally sectioned to a thickness of 5 μm, deparaffinized following standard procedures, and stained with hematoxylin and eosin (45). This procedure allows visual assessment and analysis of the entire fish structure, with emphasize on the peritoneum, liver, pancreas, and muscle fibers. Bright field histological images were taken on a Zeiss Axiolab (Carl Zeiss, Germany) with CoolSNAP image capture program (Roper Sci. Photometrics). For each specimen, a minimum of five sections were analyzed and scored (data not shown). The calculated coefficient of variation was <10% between individuals.

### Classical histology

The samples prepared in the previous step were used to evaluate the resulting histomorphology on the liver, intestine, and mesentery of the λ-CGN (3.5%) treated and control ZF. The slides were recorded on a 40x objective Olympus model BX51 (Olympus Corp., Japan) microscope coupled to a 2-times projected Q Color 3 Olympus model U-PMTVC (Olympus Corp., Japan). The program used for the photographic records was the QCapture (Q Imaging) image analysis program. Subsequently, the images obtained were treated for contrast, brightness, and focus adjustment using Adobe Photoshop CC 2017.

### Plasma obtention

Blood was collected from each zebrafish using a novel analytical method (46), with slight modifications. Briefly, zebrafish were euthanized in ice-water, and the caudal fin was amputated using a pair of fine scissors. With the wound pointing down, each fish was placed in an improvised 0.5 ml microcentrifuge tube (Eppendorf, Hamburg, Germany) that had been previously perforated. The tube containing the amputated fish was placed into a 1.5 ml microcentrifuge tube (Eppendorf, Hamburg, Germany) with 10 μl of heparin (500 IU/ml). The assembly was then placed into a benchtop centrifuge (Eppendorf, Hamburg, Germany) and run at 40 x *g* for 5 min at 11°C. A new cut was made to each tail, and the centrifugation process was repeated as above. Plasma was obtained as clear supernatant after centrifugation at 12,000 x g for 10 min at 4°C.

### Protein digestion and peptide desalting

Plasma protein concentration was determined using the Bradford method (47) and precipitated by addition of cold acetone/methanol (4:1, v/v) following the protocol of Bian et al. (48), with slight modifications. Briefly, a total of 30 µg of protein was mixed with a solution of 6 M urea buffer and reduced with 5 mM DTT at 65°C for 60 min and alkylated by addition of 15 mM iodoacetamide (IAA) at 65°C for 60 min in the dark. Digestion of the proteins was performed by adding proteomic grade trypsin (Sigma, USA) at a protease-to-protein ratio of 1:100 (w/w) followed by incubation for 18 h. After desalting with Zip-Tip (Waters, Milford, MA, USA), peptide samples were dried in a SpeedVac vacuum concentrator (Thermo Fisher Scientific, USA). Finally, samples were eluted in 20 µL of 0.1% formic acid before analysis by nanoflow liquid chromatography/tandem mass spectrometry (LC−MS/MS).

### Shotgun LC-MS/MS

The poled plasma samples from nine fish were analyzed in triplicate using a hybrid linear ion trap–Orbitrap mass spectrometer (LTQ-OrbitrapVelos Pro; Thermo Scientific, Waltham, MA, USA) equipped with a nano-electrospray ionization source, as previously described (49). Online reversed-phase HPLC was performed with an Easy nanoLC system (Thermo Fisher Scientific, USA). Triplicates of each sample (5 µL) of the resulting peptide mixture were injected into a trap column packed with C18 (100 µm i.d. × 2 cm) for desalting with 100% solvent A (0.1% formic acid). The peptides were then eluted into an analytical column (75 µm i.d. × 100 mm) packed in house with Aqua^®^ C-18 5 µm beads (Phenomenex, USA). Elution of tryptic peptides was performed, in a column, in a 120 min linear gradient from 3 to 50% of solvent B (acetonitrile in 0.1% formic acid) at a flow rate of 200 nL/min. Spray voltage was set at 2.1kV, at 200°C and the mass spectrometer was operated in a data-dependent mode, in which a full MS scan was acquired in the m/z range of 300-1650, followed by MS/MS acquisition using Collisional Induced Dissociation (CID) of the 15 most intense ions from the MS scan. MS spectra were acquired with a resolution of 60,000 (at m/z 400) in the Orbitrap analyzer. Dynamic exclusion was defined by a list size of 500 features and an exclusion duration of 60 s (50). For the survey (MS) scan AGC target value of 1,000,000 was set, whereas the AGC target value for the fragment ion (MS/MS) spectra was set at 10,000 ions. The lower threshold for targeting precursor ions in the MS scans was 3,000 counts.

### Proteomic data processing and bioinformatic analysis

Each MS raw file was processed using ProteoWizard to generate a.mgf file. The generated files were processed in SearchGUI (version 2.7.1), which runs the search engines Open Mass Spectrometry Search Algorithm (OMSSA), MS-GF+, and X!Tandem (51). The database used for identification was the Target-Decoy version of the *D. rerio* protein database (UniProt release 09_2018; 58,895 reviewed sequences from *D. rerio* proteins) combined with common contaminants concatenated with the inverted versions of all sequences. The search parameters used were peptide and fragment ion mass accuracy 10 ppm and 0.5 Da, respectively; protein and peptide FDRs 1%; two miss cleavages; trypsin as enzyme; fixed modifications: cysteine carbamidomethylation; variable modifications: methionine oxidation. To generate the proteome dataset of the *D. rerio* samples, the resulting data were processed in PeptideShaker (52). Concentrations of detected proteins were estimated using the weight fraction (weight %) of each protein in plasma obtained based on its emPAI (exponentially modified protein abundance index) score in the MS profile and its molecular weight (53). Quality control filtering was performed with 1% FDR. Data from three technical replicates were pooled together. (Raw data are available in MassIVE https://massive.ucsd.edu, accession number MSV000088678).

### Ontology, pathway network analyses, and structural 3D images comparisons

The zebrafish and human proteins were retrieved from the ENSEMBL ontological annotations, and the interacted proteins were subjected to functional enrichment using G:Profiler (54). Next, we used the Gene ontology (GO) and Kyoto encyclopedia of genes and genomes (KEGG) enrichment analysis to evaluate the function and identify the pathways. The identified proteins were blasted against *D. rerio*, and the protein-protein interaction (PPI) networks were mapped using the Search Tool for the Retrieval of Interacting Genes/proteins (STRING) database (55). The pathway-enriched genes originated from the open-data resource of human pathways and reactions database, the Reactome (56). Eventually, we calculated the similarity percentage between the zebrafish and human putative orthologous proteins using EMBOSS and performed the protein alignments with ESPript. Then, we produced structural images of proteins with the PyMOL (57). To compare the 3D structures, the FASTA files were converted into PDB files using Raptor X, and the structural similarities were compared using the iPDA platform.

### Statistical analysis

Normality of the dataset was tested by the Shapiro-Wilk test and the significance between different groups was determined by analysis of variance (ANOVA) using a completely randomized split-plot design with subunits in time. Unpaired t-test for two groups and multiple comparisons and *post hoc* Tukey’s test for extended groups were applied. Statistical significance was set at (*p < 0.05*). All statistical analyses were performed in R (58), and the figures presented were produced using the package ggplot (59).

## Results and discussion

### λ-CGN induces abdominal edema formation in zebrafish

**Figure 1:**
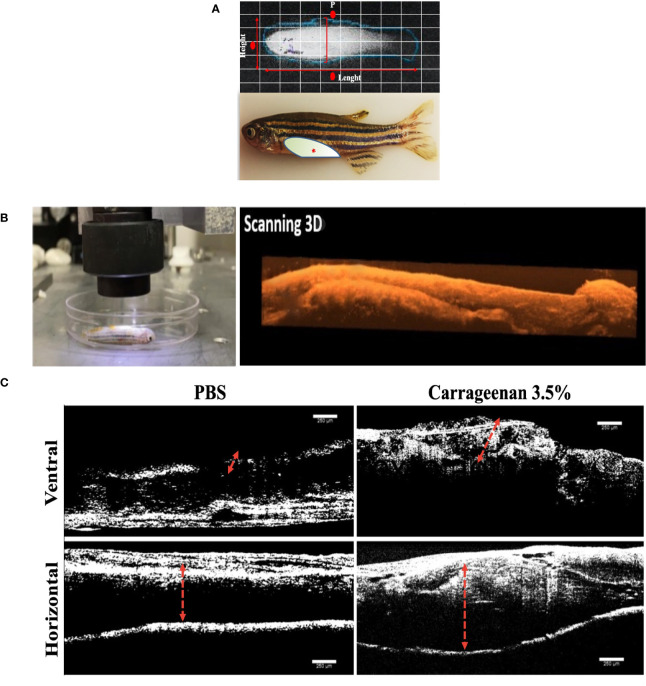
Characterization of the λ-CGN-induced abdominal edema model of inflammation in zebrafish (*Danio rerio*) male adults. **(A)** Representative images of healthy adult zebrafish at the beginning of the trial. (*Upper panel*) *In vivo* X-ray image (Xtreme system). (*Lower panel*) Real-time image of live zebrafish showing the λ-CGN injection site (red asterisk). The light color represents the abdominal cavity area forming edema analyzed in this study. **(B)** Live immobilized λ-CGN-induced male zebrafish specimens were evaluated one by one following a side and ventral pattern using a classical optical coherence tomography (OCT) device. The representative scanning 3D reconstruction is presented on the right side. **(C)** Spectral domain OCT images of a healthy and inflamed fish highlighting the edema boundaries and tissue disorganization (red dotted lines) on the ventral and horizontal superficial layers 4h post-injection of PBS or 3.5% λ-CGN, respectively. Scale bar 250 µm.

To characterize the physiological response of inducing an inflammatory process with type IV λ-CGN in adult zebrafish, we injected i.p. (**Figure 1A**) twenty animals per treatment group with three different dilutions 0, 1.75, and 3.5% (v/w). We found similar dose-dependent inflammatory reactions in the coelomic cavity, expressed as prospective peritoneal edema between the different treated groups. Changes between inflammation and control conditions were assessed in each fish in real-time *in vivo*, and 3D reconstructions were obtained using classical optical coherence tomography (OCT) (**Figure 1B**). The spectral domain of OCT highlighted the edema boundaries and tissue disorganization. It revealed the highest visual damage score in the λ-CGN administered groups, independent of dose after 4h of treatment. For a clear visual perspective of the OCT results, we present representative images of ventral and horizontal perspectives obtained for the PBS control (0%) and the highest λ-CGN (3.5%) treated dose (**Figure 1C**). The OCT technology has previously been demonstrated as a powerful method to assess reflectivity caused by plasma infiltration in numerous animal models. For example, in support of the specificity and accuracy of OCT technology, Sonoda et al. in 2014, demonstrated the general physical changes and analyzed the reflectivity of specific components infiltrating into the chambers of a swine eye (60). Furthermore, our findings support those of Haindle et al., who recently suggested OCT technology as a fast-scanning, non-invasive, and label-free multimodal ultra-high resolution imaging system to reveal morphological, pathophysiological, and drug responses using *in vivo* zebrafish specimens of various developmental stages (61). Although considerably simplistic, our λ-CGN peritoneal inflammation model in zebrafish was successfully established at this stage, limited to characterizing the relative intensity with which fish tissue physically reacts to induction with the inflammatory agent.

### λ-CGN stimulates a selective cellular immune response in zebrafish

**Figure 2:**
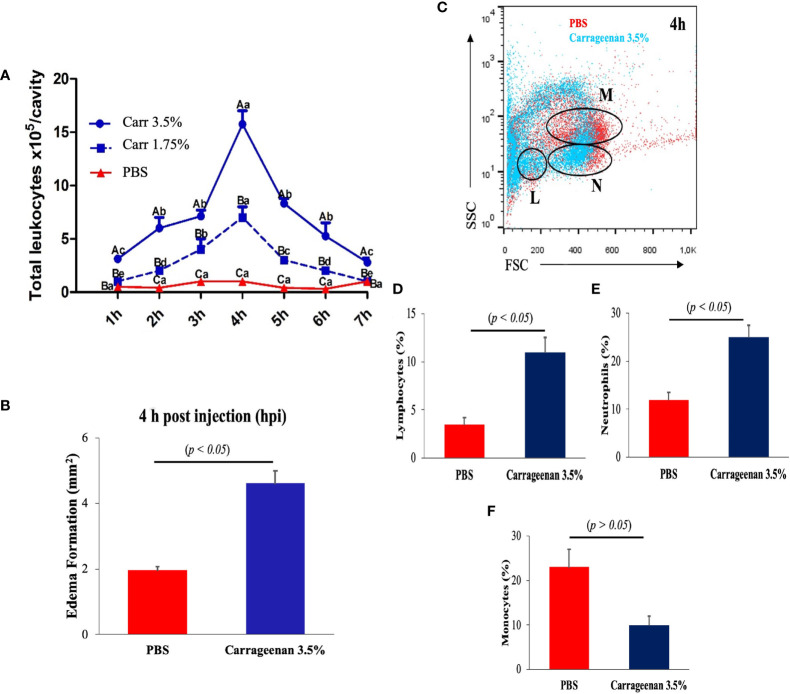
Intraperitoneal injection of λ-CGN stimulates a highly selective cellular immune response in zebrafish. **(A)** The time-dependent infiltrating mean number of total leukocytes in control (PBS) and λ-CGN-injected fish with two different concentrations. **(B)** Quantification of the progressive mean abdominal edema expansion using the highest (3.5%) λ-CGN concentration 4h after the injection. **(C)** Representative dot plot showing the gating used to discriminate between lymphocytes [L], neutrophils [N], and monocytes [M] present in the ascites recovered from the PBS (red) or 3.5% λ-CGN-treated (blue) fish 4h post-injection. **(D–F)** Quantification of data in **(C)** by cellular type. (Note the resulting inhibition in the monocyte fraction) (n = 21 fish per group) Results were analyzed using an unpaired t-test (*p*< 0.05) respective to the control value. Error bars indicate SD.

Next, to reveal the cellular mediators leading the response in the model, aliquots of the resulting peritoneal edemas were collected hourly from each λ-CGN-treated fish per group along a 7h period and analyzed by flow cytometry. Both concentrations of λ-CGN-treated groups showed significant peritoneal edema. Light microscopy analyses of the edema content revealed that leukocytes were the most abundant cell type infiltrating the liquid phase of the edema. However, at 4 h post-treatment, we recorded an exceptionally high value for the 3.5% λ-CGN group, which significantly (*p*< 0.05) triplicated the value of the PBS control group (**Figure 2A**). Consistent with our work, Huang et al. in 2014 reported that λ-CGN at 3.0% (v/w) injected i.p. in adult zebrafish induced abdominal edema and correlated protein expression of myeloid-specific peroxidase (Mpx), tumor necrosis factor-a (Tnfa), and nitric oxide synthase (iNos) as mediators of the inflammation (14). More recently, using a similar setting based on adult zebrafish stimulated for 4h with a high dose (3.5%) of λ-CGN and following a short transcriptomic approach, Belo et al., 2021, described the kinetics of the proinflammatory (*ilib*, *tnfa*, *nos2*, *nrf2*) gene expression (27). Besides, the same authors examined the histochemical activity of Tnfa, Il1b, Nos2, and Mhc2 proteins in the liver, spleen, and kidney of the fish treated with λ-CGN. Surprisingly, these authors found that renal tubules and vascular endothelium were the tissues displaying the highest immunoreactivity. Regardless, questions about the cellular composition of zebrafish and the activities that follow the encounter with λ-CGN remain controversial. Recent studies in mice have suggested macrophages as mediators of inflammation. Moreover, the mechanisms by which macrophages acquire λ-CGN in the lumen at the steady-state and trigger inflammation have been speculated (62). Therefore, based on several reports on mammalian models, we hypothesized that acute inflammation in zebrafish induced by λ-CGN might change the kinetics of monocyte differentiation while increasing the number of macrophages infiltrating the gut to provide tissue protection during injury (63, 64). To answer this hypothesis, we carefully obtained from the peritoneal edema the liquid phase generated in each fish by the λ-CGN injection. Each suspension was run through a classical flow cytometer protocol where side and forward scatters were analyzed to determine changes in the leukocyte population between treatment and control conditions (**Figure 2B**). Moreover, each aliquot of the analyzed cell suspension was loaded into a Cytospin chamber, stained with May-Grüenwald Giemsa, fixed and characterized by light microscopy following standard morphological criteria. Furthermore, these results were supported by the morphological characterization and optic counts of the treated cell aliquots used in the FACS (Data not shown). The flow cytometry dot-plot analysis overlapping both conditions (PBS vs. Carrageenan 3.5%) revealed in the λ-CGN treated animals a considerable accumulation of lymphocytes (L) and polymorphonucleated cells (N) (**Figures 2C–E**), dominated mainly by neutrophils, as determined by classical microscopy analysis. However, strikingly, the number of monocytes was inversely correlated, suggesting a partial inhibition by the treatment (**Figure 2F**). Mononuclear phagocytes, including monocytes, have been considered the primary innate immune cells. However, as in our study, in chicken and mice, monocyte depletion has been observed after *in vivo* carrageenan treatment (65, 66). These similarities suggest that a monocyte/macrophage depletion by the λ-CGN treated host may be a potentially beneficial strategy to significantly reduce inflammation if the engagement of activated macrophages with T-cells to produce exacerbated Th responses is interrupted. Yet, the mechanisms that allow for such context-dependent outcomes have not been fully defined.

### Histopathological features in whole-zebrafish mounts treated with λ-CGN

**Figure 3:**
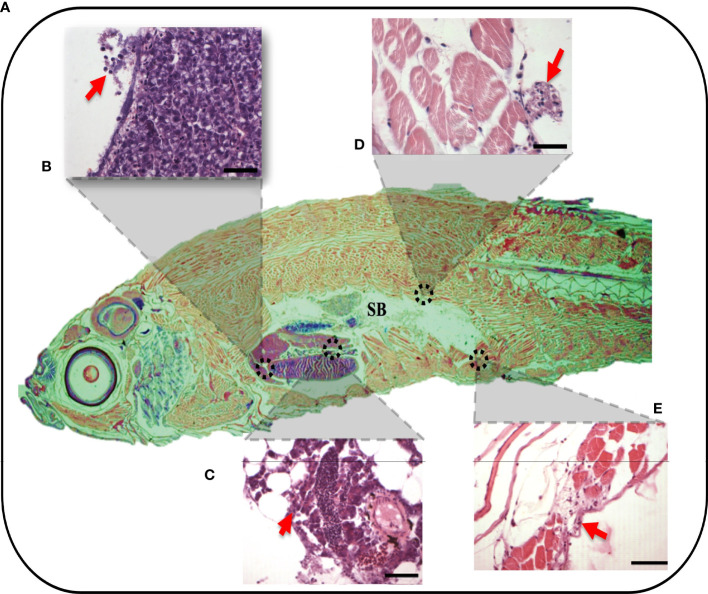
Whole organism imaging of H&E-stained adult zebrafish enables the visualization of histology-like cross-sections. **(A)** Representative sagittal cross-section of adult wild-type zebrafish resulting after 4h post-injection of 3.5% λ-CGN by i.p. **(B–E)** The histopathological lesions recorded on selected tissues. **(B)** The projection showing detail of liver damage with the arrow indicating a leukocytes infiltration in the hepatic fracture caused by the induced swelling. **(C)** Pancreas with inflammation and recruited leukocytes present in the connective tissue capsule. **(D)** Dorsal muscle fibers at the edge of the peritoneal cavity showing muscular edema, the arrows highlight the infiltrating cells. **(E)** Ventral muscles denoting a clear inflammatory process on top of the muscularis and besides muscle packs. (n = 10). For reference the swim bladder-SB is indicated. Scale bar 50 µm.

To validate these findings, the migratory behaviors of leukocytes along and beyond the peritoneal cavity and major organs were assayed in the context of the whole organisms treated with 3.5% λ-CGN using classical histopathological techniques. Previously, cellular composition and tissue architecture have been extensively described in zebrafish by applying histological techniques, while average and abnormal cytological features indicative of physical, neoplastic, and inflammatory pathologies have been differentiated (67). In the present study, λ-CGN-exposed whole animals were formalin-fixed and paraffin-embedded to obtain representative sagittal cross-sections to allow histopathological evaluation of each organ without losing the perspective of the entire animal (**Figure 3A**). It is important to note that decalcification in acid-base was not used to avoid tissue destruction or to obtain pale staining, as previously reported (68). Reactive PMN’s were recorded throughout the peritoneal cavity with a multifocal pattern in the conjunctive and parenchymal tissues. A key feature of inflammatory cells is the ability to migrate to a site of injury or infection quickly and efficiently (69). In particular, the migration of leukocytes from the peritoneal edema into vital organs such as the liver (**Figure 3B**) and pancreas (**Figure 3C**), or essential conformational tissue like the dorsal and the ventral muscle fibers (**Figures 3D, E** **)** located at the edge of the peritoneum was revealed. In addition, a separate analysis revealed a modest number of mononuclear monocyte-like cells adhered to the cavity walls, which were associated with exudates. Our results are consistent with those obtained in mice where total leukocyte recruitment significantly increased, with a predominance of neutrophils. However, no mononuclear cells were observed in the peritoneum and all adjacent tissues within a few hours of receiving λ-CGN by injection (70). In our model, histopathological lesions affecting key organs contained in the peritoneum of the inflamed zebrafish achieved a threshold at 4h after injection. Interestingly, all tissues analyzed showed moderate but evident infiltration of leukocyte clusters, suggesting a primary role of immunocytes in the inflammatory reaction. This behavior was expected, as chemical induction of inflammation in zebrafish has previously been linked to the overexpression of PMN leukocytes, particularly the most abundant type, the neutrophils (71). Our results collectively conceive the peritoneum as an organ with lymphoid characteristics, which causes migration and stimulation of the immune cell, leading to powerful inflammatory reactions as previously suggested in mammals (72) and, more recently, in fish (73).

### λ-CGN alters the zebrafish peritoneal mucosal architecture by strongly recruiting polyreactive leukocytes

**Figure 4:**
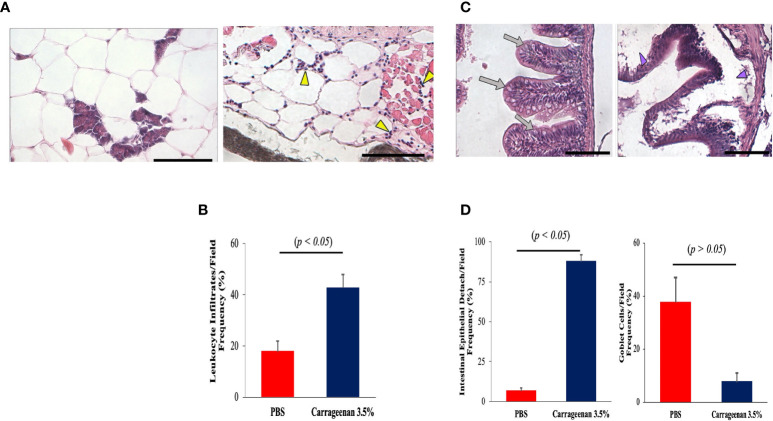
The inflammatory condition caused by λ-CGN extends along the entire boundaries of the peritoneal cavity. **(A, B)** Mesentery. (A-left) Basal condition showing the absence of reactive leukocytes in the mesenteric conjunction between the adipose cells and the pancreatic tissue. (A-right) In the mesentery of the 3.5% λ-CGN-induced swollen fish, extensive leukocyte infiltrates are observed after 4h. Note the vast leukocyte (yellow arrowheads) infiltrating the area and into the adjacent musculature. **(B)** Graphical representation of the leukocyte infiltrate counts per optical field (n = 10) in the λ-CGN treated fish. **(C, D)** Intestine. (C-left) Basal condition showing the unaltered physiological architecture of the intestine where epithelial cells, goblet cells and intestinal folds are clearly defined (gray arrows). (C-right) In the fish treated with 3.5% λ-CGN, obvious histopathological alterations and a strong reduction of fish intestinal loops and major structural components due to fluid accumulation (edema) are clearly observed (purple arrowheads). **(D)** Percentage quantification of the intestinal epithelia detachment (Left) and Goblet cells (Right) recorded in 10 fields per slide (n = 15 slides per condition) between the control and the λ-CGN-treated fish. Sections stained with hematoxylin-eosin. Objectives 40X; projective 2X. Staining: regular H&E-Harris. Scale bar 80μm at 40X. Results were analyzed using an unpaired t-test (p < 0.05) respective to the control value.Error bars indicate SD.

Next, we attempted to cross-examine the adjacent peritoneal tissues, namely the connective mesentery, parenchyma, and intestine in the λ-CGN-induced and control groups. Unlike the previous experimental reports available using the λ-CGN inflammation model, only the present investigation reported on formation of edema and hyperemia damage induced in the mesentery as the main foci of polyreactive cells surrounding endocrine tissue (**Figure 4A**) and the intestinal loops (**Figure 4C**). In addition, we also demonstrated leukocyte infiltration in the mesentery (**Figure 4B**) and edematous epithelial detachment and goblet cells in the most affected intestinal tissue (**Figure 4D**) of the λ-CGN treated fish. These data are consistent with a recent *in vivo* report using rats. Pogozhykh et al. in 2021, evaluated food-grade semi-refined carrageenan toxicity and observed it in the small and large intestines of treated rats, a high leukocytes infiltration and fewer numbers of goblet cells and consequently, mucin production in this experimental group was less pronounced than in the control (74). Furthermore, it is well known that PMN leukocytes and their effector molecules can function as a double edged-sword promoting tissue injury and contributing to tissue homeostasis (75). Thus, our findings also revealed that the presence of λ-CGN in the peritoneum of fish produced a transient presence of polyreactive leukocytes. However, their short activation is enough to produce edematous epithelial detachment in the intestinal loops in all treated fish, with more evident histopathology among the more sensitive animals. In addition, portions of the intestinal tunica were infiltrated with granular cells and revealed with classical staining techniques (data not shown). Several previous reports using different vertebrates have associated an excessive leukocyte infiltration with marked inflammatory patterns that are solely mediated by the release of potent soluble mediators contained in the granules of polyreactive immunocytes (76, 77). In the present study, the increased number of leukocytes in animals exposed to λ-CGN produced a strong inflammatory response and consistently adhered to the mesentery and serosal surface of the entire body cavity surrounded by fibrous connective and endocrine tissues.

### Overview of λ-CGN-induced inflammation in zebrafish plasma protein functions

**Figure 5:**
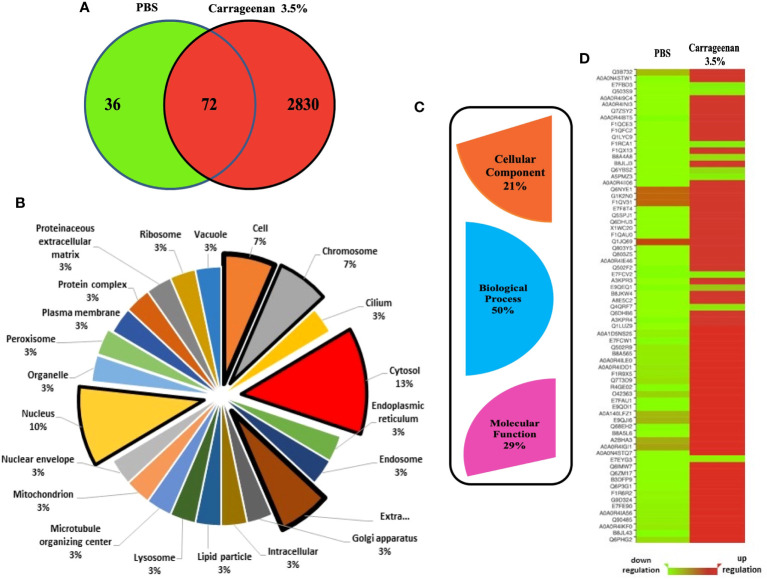
Analysis of proteomics data. **(A)** Venn diagram created using the program Venny (http://bioinfogp.cnb.csic.es/tools/venny/index.html) showing the total number of proteins differentially expressed after 4h between the PBS and λ-CGN (3.5%) injected fish. Seventy-two shared proteins between the three replicates analyzed per group were further analyzed. Following identification, groupings were done according to important proteins in each set involved in the process of λ-CGN-induced inflammatory reaction in zebrafish. Protein interactions were also investigated with regards to their **(B)** Cellular component and **(C)** GO Domain using the STRING software (version 5.18). **(D)** Heatmap showing the raw data (score) of the 72 proteins identified in plasma extracted from the PBS and λ-CGN induced fish. Note the strong protein differential expression achieved in the λ-CGN treated zebrafish. The visual guide is located at the bottom.

To determine the systemic impact of peritoneal inflammation on λ-CGN induced inflammation in zebrafish, we investigated alterations in the plasma protein profile. To this end, digested plasma protein samples were analyzed using an optimized reversed-phase high-performance liquid chromatography system (LC) to separate peptides, followed by a hybrid linear ion trap mass spectrometry (MS)/MS shotgun proteomic approach. Outputs consistently demonstrated that the λ-CGN induced inflammation resulted in a larger plasma proteome. A total of 2938 differentially expressed proteins were identified in both conditions. Only a relatively small number of 108 proteins were recorded in the control group. Thus, the number of differentially expressed proteins (2902) identified in the λ-CGN treated group was considerably higher than in the control (**Figure 5A**). After differential expression analysis, the complete set of proteins obtained was grouped according to their relevant qualitative cellular component. Twenty-two different locations were recorded (**Figure 5B**). The use of biosynthesized artificial proteins composed of tandemly connected peptides resulting from the comparison of the induced inflammatory process has previously been shown to be a valuable strategy for quantitative analysis of multiple proteins by LC-MS/MS (78). Interestingly, in the present study, out of the 22 different locations, two environments, the nucleus and cytosol, hosted 23% of the total recorded proteins. Further, they were mapped for their cellular component, biological processes, and molecular functions based on the STRING gene ontology (GO) analysis to predict the biological consequence of differentially regulated plasma proteome. Examination of the identified proteins by GO enrichment revealed that the total blood proteome was encompassed by three main functional processes. Cellular component (21%), molecular function (29%), and the highest represented biological process dominating 50% of the predictions (**Figure 5C**). A total of 72 common proteins detected in both the control and 3.5% λ-CGN groups were significantly (*p* < 0.05) differentially regulated between them. Of the commonly expressed proteins with extended relationships, compared to the control group 62 proteins got a significant (*p* < 0.05) upregulation in the λ-CGN-treated group, while the remaining ten proteins were downregulated. Interestingly, APPs can be classified as positive or negative, depending on their serum concentration during inflammation (79). Negative APPs are downregulated, and their concentration decrease as a homeostatic response of the host during an inflammatory event. In our setting, the differential expression of the 72 recorded proteins is presented as a heatmap of the raw data obtained for both groups (**Figure 5D**). Several studies using λ-CGN as a model of local inflammation in various vertebrate animals have shown that the use of this substance causes localized edema, infiltration of leukocytes, and increased levels of inflammation (80–82). However, the associated mechanisms remain elusive. Several studies have contributed to fill the gap using transcriptomic approaches, but only a few have provided evidence at the functional protein level, as we do in the present study. An attractive recent *in vitro* trial using whole blood cultures from Yorkshire barrows treated with λ-CGN illustrates the differences in these two omics levels. The authors found that alveolar macrophage-derived neutrophil chemotactic factor-II (AMCF-II), a swine-specific member of the IL8/GRO family, showed an increase in gene expression but not at the protein level (83). In contrast, the effect of λ-CGN on IL-8, a critical proinflammatory and chemotactic cytokine in the recruitment of neutrophils, at the protein level produced a high expression but had no impact on the same level of a directly related molecule, the serum amyloid A (SAA). Therefore, the ability of λ-CGN to significantly mediate the transcription and translation of closely related molecular functions suggests that careful analysis applies when dissecting its role in modulating inflammation and immune responses.

### Biological context and interactome analysis of the most abundant plasma proteins in λ-CGN-inflamed zebrafish

**Figure 6:**
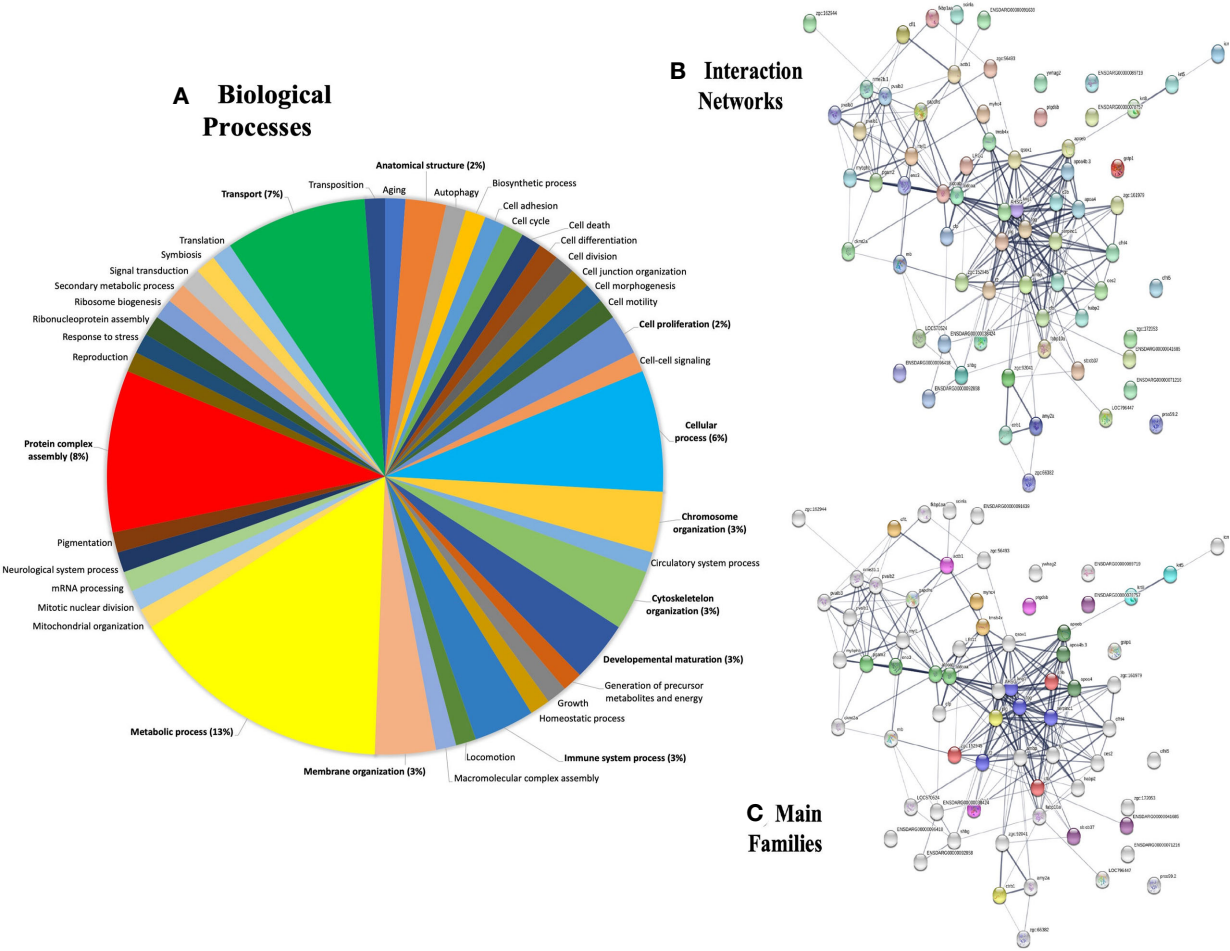
Gene ontology (GO) annotations retrieved from plasma proteins in the zebrafish λ-CGN-induced inflammatory reaction after 4h. **(A)** The forty-two differentially expressed genes were involved in biological processes, mainly in metabolic process (13%) and transport (7%) as the top terms. **(B)** Major protein-protein interaction network among hub protein clusters in the blood plasma of λ-CGN-induced fish. The stronger associations are represented with thicker lines. **(C)** Functional pathway network showing the distribution of pathways associated with the complement system and blood coagulation cascade identified through the functional enrichment analysis of λ-CGN-treated fish. The color depth of nodes refers to the corrected *p-value* of the ontologies represented: (purple, immunoglobulins; green, apolipoproteins; orange, complement; blue, fibrinogen). Hierarchical clustering of hub protein-protein interaction networks functional enrichment analysis was constructed using STRING (https://string-db.org). The associations between the twenty-seven proteins of this signaling pathway were predicted using the KEGG tool. Names and taxonomy retrieved from UniProtKB (See **Table 1** for accession numbers): Keratin 5 (Krt5); Keratin 8 (Krt8); Cofilin 1 (Cfl1); Myosin heavy chain 4 (Myhc4); Actin (Actb1); Retinol binding protein 4 (Rbp4); Alpha-2-macroglobulin-like protein (A2ml); Alpha-2-macroglobulin-like 1 (Si:dkey-105h12.2); Alpha-2-macroglobulin (Sb:cb37); Apolipoprotein Eb (Apoeb); Apolipoprotein A-IV b,2 (Apoa4b.2); Apolipoprotein A-IV b,1 (Apoa4b.1); Phosphoglycerate mutase (Pgam2); Phosphopyruvate hydratase (Eno1); Fructose-bisphosphate aldolase, b (Aldoab); Fructose-bisphosphate aldolase, a (Aldoaa); Kininogen 1 (Kng1); Fibrinogen gamma chain precursor (Fgg); Antithrombin-III precursor (Serpinc1); Prothrombin (F2); Complement C3a, tandem 2 (C3a.2); Hemopexin (Zgc:152945-001); Complement factor B (Cfb); Plasminogen (Plg); and, Chymotrypsinogen B,1 (Ctrb.1).

To achieve this goal, the annotation deposited in the Gene Ontology (GO) database was retrieved and the major protein-protein interaction networks for zebrafish were obtained. The GO distributions obtained for biological processes are presented here (**Figure 6A**). From the proteins found in plasma after the λ-CGN-induced inflammatory reaction in zebrafish, the 27 most abundant proteins were identified and statistically validated through the bioinformatics tool Mascot Distiller (www.matrixscience.com) and STRING software where interaction networks and main families were obtained (**Figures 6B, C**). Previous research has established that the basal zebrafish and human plasma proteome share significant similarities (84). Essentially, all identified proteins retrieved from UniProtKB, (**Table 1**) correspond to important inflammatory pathways. Therefore, the recorded behavior for most of the identified proteins was expected. Concomitant to the λ-CGN induction of inflammation, PMN leukocytes and lymphocytes rapidly migrate and strengthen, promoting the production of several pro-inflammatory mediators through different complementary pathways, and inducing polyreactive synthesis granulocytes extravasation into the focal target area (85–87), in this case the peritoneum. Therefore, the present results suggest that despite λ-CGN being considered a dietary-grade element, it is a potent inflammatory mediator with enough power to trigger varied and uncontrolled protein mediators that may lead to possible detrimental effects associated with the use of λ-CGN in Western and fast-food diets for human consumption.

**Table 1:** Retrieved details for the 27 most abundant proteins identified in zebrafish plasma after 4h of the λ-CGN-induced inflammatory reaction.

#	Protein name	Description	Entry
1	Krt 8	Keratin, type II cytoskeletal 8	Q6NWF6
2	Krt 5	Keratin 5	E9QBD7
3	Cfl1	Cofilin 1 (non-muscle), like	Q7ZWD8
4	Myhcy4	Myosin heavy chain 4	A2BGX6
5	Tmsb4x	Beta thymosin-like protein	Q45QT2
6	Actb1	Actin, cytoplasmic 1	Q7ZV17
7	Ptgdsb	Lipocalin-type prostaglandin D synthase-like protein	Q8QGV5
8	Rbp4	Retinol binding protein 4	Q9PT95
9	Adml	Alpha-2-macroglobulin-like protein	B8QS14
10	Si:dkey-105h12.2	Alpha-2-macroglobulin-like 1	X1WBT0
11	Sb:cb37	Alpha-2-macroglobulin	F1R8N2
12	Apoeb	Apolipoprotein Eb	E9QBB8
13	Apoa4b.2	Apolipoprotein A-IV b,2	F1QJD1
14	Apoa4b.1	Apolipoprotein A-IV b,1	F1QHR0
15	Pgam2	Phosphoglycerate mutase	Q7T3G4
16	Eno1	Phosphopyruvate hydratase	Q6TH14
17	Aldoab	Fructose-bisphosphate aldolase,	Q6P043
18	Aldoaa	Fructose-bisphosphate aldolase,	Q803Q7
19	Kng1	Kininogen 1	Q1LYJ7
20	Fgg	Fibrinogen gamma chain precursor	Q7ZVG7
21	Serpinc 1	Antithrombin-III precursor	Q8AYE3
22	F2	Prothrombin	E7FAN5
23	C3a.2	Complement C3a, tandem duplicate	F1QV29
24	Zgc : 152945	Hemopexin	A0A8M2BKS1
25	Cfb	Complement factor B	F1R886
26	Plg	Plasminogen	F1Q890
27	Ctrb1	Chymotrypsinogen B1	F1QFX9

(Source: UniProtKB; https://www.uniprot.org).

### Effect of λ-CGN on the complement system during the acute phase of inflammation in adult zebrafish

**Figure 7:**
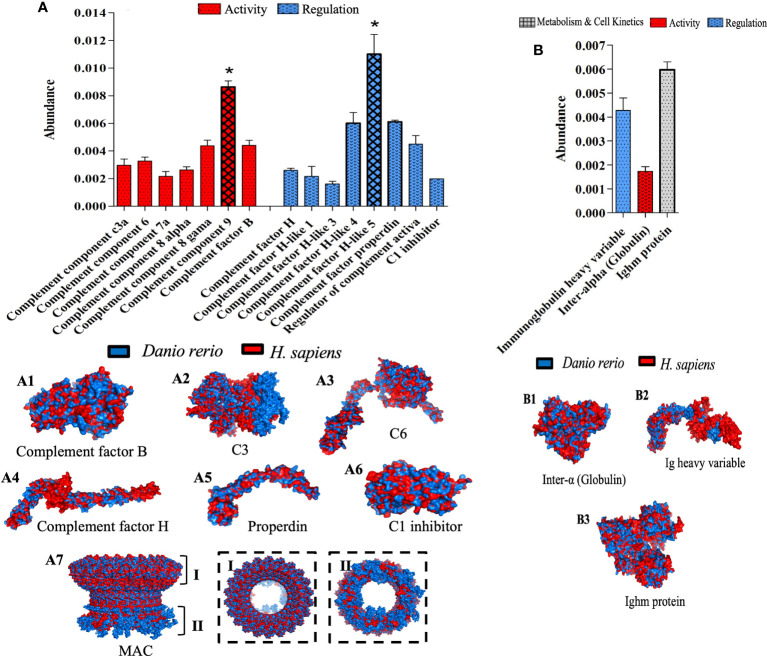
Top functional proteins linked to the complement system identified in zebrafish plasma after 4h of acute inflammation induction with λ-CGN. **(A)** The abundance of the complement component proteins quantified by their activation and regulatory patterns. (A 1-7) Representations of the structural 3-D protein complement factors expressed in zebrafish (blue) plasma after the λ-CGN-induced inflammation overlap with their predicted *Homo sapiens* (red) homologous structures. The overall architecture of the membrane attack complex (MAC). (A 7) Upper rim assembly of the barrel (I), and the cytoplasmic amphipathic region (II). **(B)** The activity, regulation, and metabolism & cell kinetics of natural antibodies affecting the classical complement pathway. (B 1-3) Exposed 3D zebrafish proteins contrasted with those of *H. sapiens*. The values in the graphs are expressed in relative abundance of the sample average (n = 4). The structural analysis of the proteins is described in the methodology. Abundance increases denoted by asterisks. Data are representative of two repeated trials in which all samples were run in triplicate (Student’s t test, p < 0.05). Results were analyzed using an unpaired t-test (p < 0.05) respective to the control value. Error bars indicate SD.

The effects of λ-CGN present in the peritoneal environment were assessed through histopathological damage and significant differential expression of several proteins, mainly those related to the biological process of immunity. Therefore, to understand the molecular underpinnings of these effects, and based on the unveiled protein sets, we performed a bioinformatic structural similarity analysis and 3D reconstructions of zebrafish treated with λ-CGN and the corresponding human counterparts. Raptor X2 generated structural similarities, and the structural overlay was analyzed in the iPDA.1. Biological pathway analysis pointed to the activation and abundance of various complement system factors. Notably, 4h post-injection of λ-CGN in the zebrafish peritoneum strongly induced after 4h the complement and downstream coagulation proteins (**Figure 7A**). These two pathways are evolutionarily related to proteins such as factor H or complement components C3a and C5 (88). Interestingly, complement and coagulation proteins are largely produced by hepatocytes (89), and we observed a strong leukocyte infiltration in the liver, adding to the speculation that λ-CGN acts in synergy with PMN cells and incites the liver to induce at least some of the alterations reported here. In our setting, we also observed that λ-CGN significantly (*p*<0.05) regulates the intermediate complement components, activators like properdin, the regulator of the active complement, or the C5downstream signaling. Moreover, the structural overlap between fish and human protein is highly correlated (**Figure 7A**; 1-6). Interestingly, neutrophils recruited to an inflammatory focus have been reported to activate by C5 and quickly release C3a, which is critical for the assembly of the complement alternative pathway, where H factors function as a stabilizer (90). When a binding site for C8 is exposed on C5, the complex inserts deep into the membrane, then C9 binds to C8 and undergoes significant conformational levels leading to the formation of the membrane attack complex (MAC) (91). The 3D structure of the MAC protein (**Figure 7A**; 7) shows the intracellular extracellular surface (I) and (II) and calcium influx channel, while the striking structural similarity of these proteins between *D. rerio* and *H. sapiens* is presented in the spotted frames on the left, respectively. At any rate, since MAC enhances the expression of E-selectin and ICAM-1 and directly stimulates the release of mitogenic substances from endothelial cells, the fine-tuning of C5-9 channel is crucial in the regulation of MAC and thus the overall function of the cell during the normal or inflamed stage. Moreover, in our setting, we speculate on activation of the alternative pathway, but also infer activation of the classical pathway. Antibody activity is demonstrated by detecting and increasing precursor proteins and IgM itself, and low active levels of the inter-alpha inhibitor. This increase may be related to activation of the classical complement pathway and direct neutralization of the λ-CGN molecule by preformed or natural antibodies to polysaccharide lipids stimulated by commensal bacteria in the gut (**Figure 7B**). Despite the structural difference in the number of chains between human tetrameric IgM and pentameric teleost fish. When compared, the full set of overlapping proteins, without losing the perspective that IGHV is species-specific, overall, they showed high levels of similarity between fish and humans (**Figure 7B**; 1-3). In summary, the differences in the inflammatory processes observed between treated and non-treated fish and the direct effects of the λ-CGN on the plasma complement and coagulation pathways partially explain some of the differential protein expressions recorded above.

### Superoxide dismutase and lysozyme orchestrate the acute inflammation and tissue injury induced by λ-CGN

**Figure 8:**
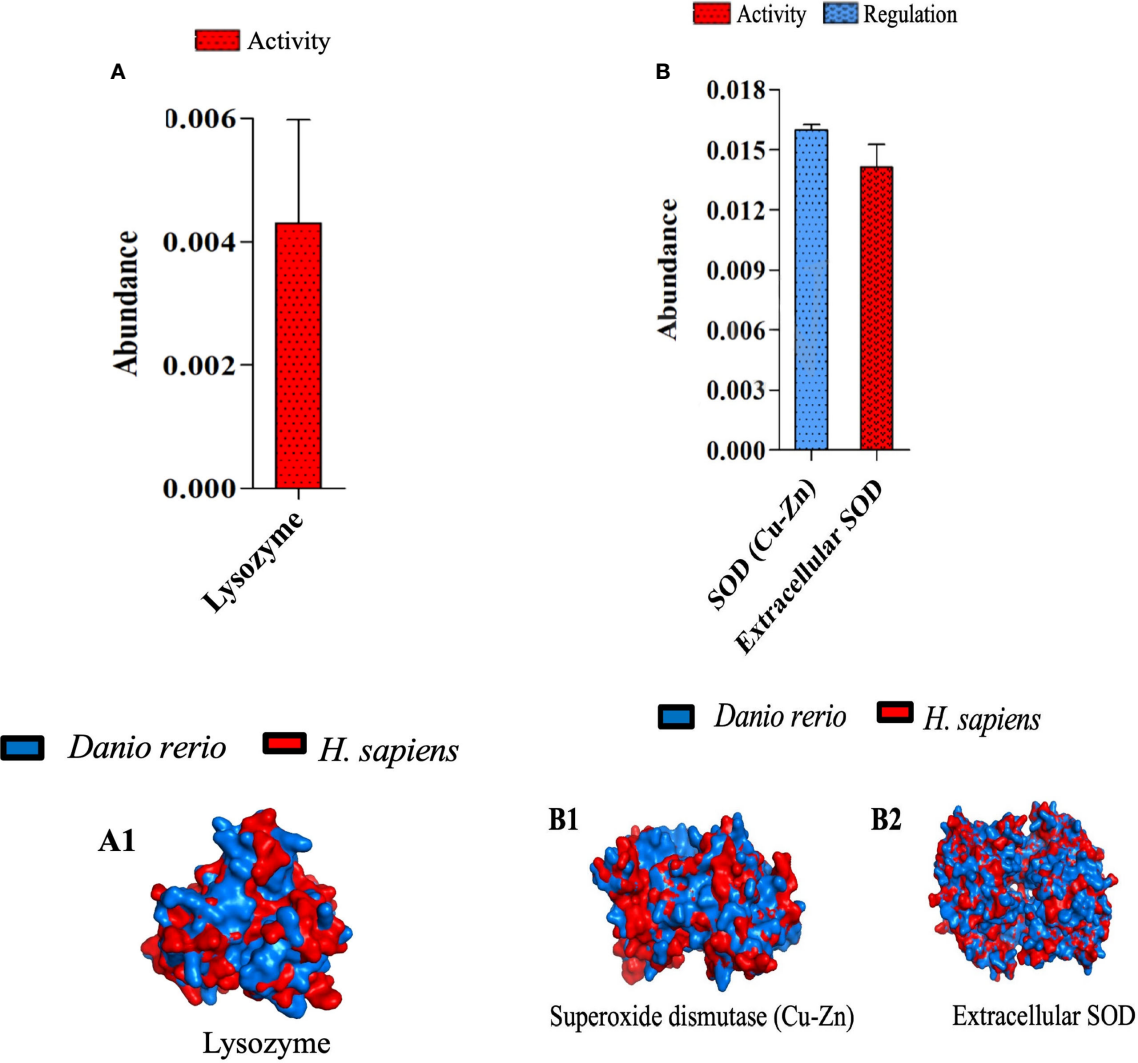
Characterization of three zebrafish orthologues of human catalytic enzymes participating in inflammatory processes mediated by λ-CGN. **(A)** The abundance of the intra- and extra-cellular superoxide dismutase (SOD) production and its role in zebrafish physiology, 4h post-injection with 3.5% λ-CGN. (A 1-2) Representations of the structural 3-D proteins in zebrafish (blue) plasma overlapping with their predicted *Homo sapiens* (red) homologous structures. **(B)** the abundance of the Lysozyme activity following the same setting as for SOD. (B 1) 3-D protein in zebrafish (blue) plasma overlapping with its predicted *Homo sapiens* (red) homologous structure. The values in the graphs are expressed as relative abundance of the sample average (n = 4). The structural analysis was obtained as described in the methodology.

To determine the effects of central immunogenic enzymes in our model of acute inflammation, we analyzed the resulting proteome of lysozyme and superoxide dismutase (SOD) 4h after receiving the λ-CGN injection. In addition, we explored their usefulness as possible markers of PMN cells activation. Lysozyme expression in the plasma of inflamed fish was found to be abundant and substantially active (**Figure 8A**). Lysozyme has been well recognized in all three types of neutrophil granules and is therefore used as a useful marker of myelopoietic activity in both zebrafish and humans (82, 83). Similarly, the extracellular SOD fraction follows a similar pattern to that recorded for Lysozyme. However, the intracellular fraction (SOD Cu-Zn) also showed a high abundance but marked biological regulatory activity (**Figure 8B**). Generally, the expression of Cu-Zn SOD is induced by shear stress and hyperoxia (79). In contrast, EC SOD, is triggered by a wide variety of inflammatory cytokines (80). Consistent with these findings, the plasma proteins analysis revealed the ascites present in the edema formed as a result from the λ-CGN injection together with regular blood flow, are candidates for the neutrophilic source of shear stress and a tensile stretch originating from rolling. Therefore, given that SOD has antioxidant enzyme scavengers for reactive oxygen species (ROS), our findings support the possible adverse effects that may result from using λ-CGN in human dietary products. Remarkably, Cu-Zn SOD also showed robust regulatory activity. Cu-Zn SOD is an effective agent to control neutrophil-mediated inflammation acting *via* the induction of apoptotic mechanisms (81). We therefore hypothesize that after neutrophils depleted the λ-CGN toxic effect in the system, the Cu-Zn SOD regulatory capacity may act as a crucial determinant of the resolution of inflammation by avoiding further propagation of the internal content of dying granulocytes. Beyond the quantification of lysozyme and both forms of SOD in zebrafish subjected to the action of λ-CGN, the protein structural similarity between *Danio rerio* and the homologous protein of *Homo sapiens* were compared (**Figure 8A**; 1 & **8B**; 1-2). The results revealed substantial similarity and biological proximity between both species and thus the functional applicability of the present results in a pharmacological context.

### λ-CGN strongly activates inflammatory proteins produced by polymorphonuclear cells in the zebrafish peritoneum

**Figure 9:**
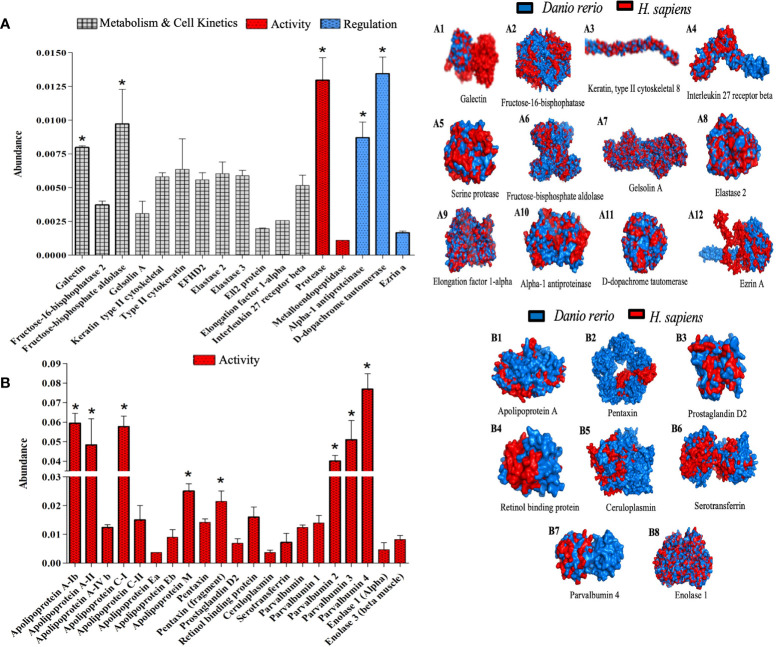
Activated polymorphonuclear leukocytes proteins orchestrate crucial inflammatory mechanisms 4h after λ-CGN injection in zebrafish. **(A)** The abundance of key proteins of cellular metabolism and kinetics, activity, and regulation is displayed. (A 1-12) Representation of the structural 3-D proteins in zebrafish (blue) plasma overlapping with their predicted *Homo sapiens* (red) homologous structures. Several inducers, mediators, and regulators of inflammation were reconstructed (E.g., Elongation factor 1-alpha, Galectin, or Fructose-bisphosphate aldolase, respectively). Note the presence and high abundance of Ezrin A in zebrafish. This protein is widely used to predict the strength of the inflammatory response in vertebrates. **(B)** The abundance of the main proteins related to the acute phase of inflammation triggered by the λ-CGN injection in zebrafish is presented. Note the marked increase in activity that was recorded in the apolipoprotein (classes: A, C, E, and M) and the parvalbumin isoforms (1, 2, 3, and 4). (B 1-8) Major circulatory proteins of the acute phase of inflammation are triggered by the main PMN cell family (Neutrophils, Eosinophils, Basophils, and Mast cells). The values in the graphs are expressed as relative abundance of the sample average (n = 4). The structural analysis of the proteins is described in the methodology. Abundance increases denoted by asterisks. Data are representative of two repeated trials in which all samples were run in triplicate (Student’s t test, p < 0.05). Error bars indicate SD.

The results so far unequivocally suggest the existence of a crosstalk between the induction of λ-CGN and PMN leukocytes as crucial effectors in the activation of the inflammatory response. PMNs perform a number of known mechanisms, including massive extravasation from the blood, release of large amounts of pre-synthesized effector proteins, generation of specialized lipid mediators, and production of a vast repertoire of rapid protein responses are conducted to accomplish systemic surveillance and containment forces against diverse damages (92). Indeed, in our setting, assessment of complement and coagulation proteins and the biological activity of immunogenic enzymes showing significant changes provide partial evidence of the mechanisms mediating the response. However, representative proteins mediating λ-CGN and inflammation engagement from the initial response to resolution and clearance are still missing. Our results further found that treatment of zebrafish with λ-CGN significantly induces chemical changes that affect the metabolic and kinetic rates of cells of many serine proteases intimately associated with PMN’s (**Figure 9A**). PMN leukocytes-restricted serine proteases possess a remarkable ability to form the inflammatory response, primarily *via* modulation of secreted inflammatory mediators (93). Some critical examples of these proteins are Gelsolin, Galectin, Ezrin, and Elastase that we found in the ZF-treated plasma proteome. Gelsolin and Galectin play an immunomodulatory role in regulating the general PMN leukocytes movement, particularly neutrophil recruitment (94, 95). At the same time, Ezrin and Elastase regulate cell adhesion by connecting critical membrane adhesion receptors, such as ICAM-I and VCAM, to the keratin or actin-based cytoskeleton in epithelial and endothelial tissues (96, 97). Interestingly, our proteomic analysis shows an increased abundance of all these proteins activation, regulation, metabolism, and cell kinetics. Moreover, extracellular proteolysis of basement membranes and matrix regulating the PMN’s diapedesis and migration are critical elements during acute inflammation. Besides, the regulatory activity of alpha-1 antiprotease and D-dopachrome tautomerase was significantly (*p*<0.05) affected. Therefore, the results propose these proteins as robust essential mechanism candidates in regulating the rampant inflammatory reaction launched by hyperactivated polyreactive leukocytes in zebrafish and similarly in humans according to the 3D overlapping (**Figure 9A**; 1-12). Likewise, as seen in figure 2A, a few hours after the inflammatory peak triggered by the λ-CGN injection, acute inflammation enters a resolution phase, perhaps induced by the secretion of anti-inflammatory cytokines by several leukocyte types that counteract tissue injury and promote healing of edema. In any case, the results suggest the practice of caution in the use of dietary λ-CGN in humans. With continued use, acute-phase proteins could directly exert a feedback loop that redirects the differentiation of the inflammatory cells to a regulatory or tolerogenic state.

Consistent with these findings, proteome analysis also revealed that some of the mechanisms involved in the APPs activation are widely abundant, supporting the inflammatory effect triggered by λ-CGN and the interaction with PMN leukocytes and perhaps lymphocytes. Interestingly, several members of the APPs are represented in our data. However, some members of the Apolipoproteins, Pentraxin, and Parvalbumin families showed highly significant (*p*<0.05) abundant levels (**Figure 9B**). Apolipoproteins are multifunctional proteins involved in cholesterol traffic and inflammatory and immune regulation through the Pparg receptor or the Arp/Nr2f2 signaling pathways (98). All members of the pentraxin family have immune regulatory functions that can act against pathogens, remove neoplastic cells, and trigger inflammation through activation of PI3K/AKT/mTOR pathways (99). Likewise, parvalbumin is a calcium-binding protein that acts through GABA and TRP receptors and can modulate inflammation through lipid mediators produced downstream of the arachidonic acid (100). Previously, we have demonstrated that TRP receptors and calcium trigger inflammation in zebrafish through a Ca2+/Tgfb-activated protein kinase pathway (101). Regardless, several other acute-phase inflammatory plasma proteins closely related to PMA’s such as Prostaglandin were abundantly expressed but not at a significant level, and the overlapping between zebrafish and humans did not fully match, which suggests a complex interplay between all mediators released by PMA leukocytes which perhaps at the mechanistic level is species-specific (**Figure 9B**; 1-8). In addition, the contribution of the liver by releasing many acute-phase molecules produced in hepatocytes could not be ruled out in this study. Enolase 1 and 3 showed a discrete abundance. Enolases increase rapidly in response to inflammatory stimuli (102). However, their expression is preferentially linked to monocytes and macrophages in mammals, but in this study, the 3D representation of Enolaze between fish and human matches at statistically significant levels. Therefore, as we previously hypothesized, the low number of monocytes/macrophages recorded in this study can perhaps be considered as a wise immune mechanism to avoid amplification of inflammatory response and thus to avoid further damage to the host due to the presence of λ-CGN in the peritoneal cavity that could further fuel the inflammatory process herein described.

## Conclusion

In conclusion, these studies demonstrate that the λ-CGN-ZF model of inflammation is effective in triggering peritoneal inflammation, and proteomics is a fully reliable procedure for quantifying the resulting changes in plasma protein abundance in the context of a whole vertebrate. By analyzing individual samples from treated and control fish, we were able to describe to a large extent the immune cells mediating the response, explore the histopathological damage produced, predict functions, and extensively characterize and quantify the resulting plasma proteome. Furthermore, we found that many critical inflammatory processes lead to the activation of several differentially expressed novel proteins in the ZF-treated group. We identified several high functional APPs related to the complement system, catalytic enzymes, and circulatory proteins. So far, however, no single protein has been shown to be the most important. Therefore, the current setting thoroughly validates ZF as a viable model of intestinal inflammation and successfully correlates *in silico* changes with homologous molecules found in their human counterparts. Although further investigation is required, the validated animal model uncovered vital proteins and inferred signaling pathways suggested in the present research that may serve as novel pharmacological targets to explore and possibly treat human inflammatory intestinal diseases.

## Data availability statement

The datasets presented in this study can be found in online repositories. The names of the repository/repositories and accession number(s) can be found below: https://massive.ucsd.edu, MSV000088678.

## Ethic statement

The animal studies were reviewed and approved by the Brazilian animal welfare legislation (CONCEA No 34, 27/07/2017 - MCTI) with prior ethical approval from the University UFMG Ethical Review Body (CEUA-UFMG 336/2017).

## Author contributions

Study conception and design: IC-S, TC, NF, AP, MB, JG-V. Data acquisition: BF, RN, FF, DM, GC, LR, MA. Proteomic data analysis: SFE, KC, ML-F, LP, DF. Histology data interpretation: JC-J, JG, MP, RB, WM. OCT data interpretation: AG, SN, AF, MR, TY. Intellectual support: TC, AN, LB, GM, JG-V. Drafted manuscript: IC-S, MB, TC, NF, AP, JG-V. All authors contributed to the article and approved the submitted version.

## Funding

This work was supported by the São Paulo Research Foundation (FAPESP, 2013/25971-9, 2019/19939-1) and National Council for Scientific and Technological Development (CNPq- 301473/2016-1). Nord University Access Fund covers the OA publication cost.

## Acknowledgments

Our thanks to Prof. Azumi Aki for the language skills provided during the writing process.

## Conflict of interest

The authors declare that the research was conducted in the absence of any commercial or financial relationships that could be construed as a potential conflict of interest.

## Publisher’s note

All claims expressed in this article are solely those of the authors and do not necessarily represent those of their affiliated organizations, or those of the publisher, the editors and the reviewers. Any product that may be evaluated in this article, or claim that may be made by its manufacturer, is not guaranteed or endorsed by the publisher.
